# Papillary Tumor of the Pineal Region Treated With Surgery and Postoperative Radiotherapy: A Case Report

**DOI:** 10.7759/cureus.77989

**Published:** 2025-01-25

**Authors:** Benjamin Royal-Preyra, Melanie Boucher

**Affiliations:** 1 Radiation Oncology, Centre Hospitalier Affilié Universitaire Régional, Trois-Rivieres, CAN

**Keywords:** neuro oncology, papillary, papillary tumor, papillary tumor of the pineal region, radiation oncology education

## Abstract

Papillary tumors of the pineal region (PTPR) are extremely rare malignancies that make up less than 0.1% of primary brain tumors. They are usually treated with surgery and adjuvant tumor bed radiotherapy (RT). We review the case of a man in his late 60s who presented with two weeks of confusion and ataxia. Imaging the head with computed tomography (CT) and magnetic resonance imaging (MRI) showed hydrocephalus and a 2 cm pineal region mass. We review the presenting symptoms, investigations, and differential diagnosis for patients with pineal region masses. The pathological features, initial hydrocephalus management, and curative treatment of his tumor with surgery and RT are discussed. We also review the PTPR literature, including prognostic features and the evidence for treatment modalities, and report adjuvant radiotherapy treatment planning volumes. The patient is symptom-free and without evidence of recurrent disease on follow-up MRI 18 months after treatment. PTPR has very high recurrence rates following treatment; less than 20% of patients have local control at 10 years, and further research is needed to find more effective interventions and improve patient outcomes.

## Introduction

The pineal gland is a small neuroendocrine organ found in vertebrates attached to the roof of the third ventricle in the middle of the brain, between the two hemispheres and outside the blood-brain barrier, which is vital for light-dark cycle regulation through melatonin secretion [1]. Tumors of the pineal gland make up less than 1% of primary brain tumors in adults and 3% in children [2]. Papillary tumors of the pineal region (PTPR) are a type of pineal parenchymal tumors that arise from the subcommissural organ's third posterior ventricle ependyma cells near the pineal gland, not the pineal gland itself [2]. The most common type of pineal gland tumors is germ cell tumors, followed by pineal parenchymal tumors that include pineocytoma (World Health Organization (WHO) grade 1), pineal parenchymal tumors of intermediate differentiation (WHO grade 2-3), PTPR (WHO grade 2-3), and pineoblastoma (WHO grade 4) [2,3]. PTPR was recognized as a distinct entity by the WHO in 2007 [4]. There is no established staging system. Less than 8% of pineal gland tumors are PTPR, under 0.08% of all primary brain tumors, making it very rare [5]. The median age at diagnosis is 29-33 years, and some studies report a slight female predominance, whereas others report a slight male predominance [2,6-7]. PTPR has a similar histologic architecture to ependymoma and choroid plexus tumors [2,6]. PTPR usually has an epithelial-type growth pattern with layers of tumor cells covering vessels and creating perivascular pseudorosettes with a similar immunohistochemical (IHC) profile to choroid plexus tumors [6]. A review of the IHC features of 31 cases of PTPR found positive staining for vimentin (VIM), neuron-specific enolase (NSE), cytokeratin (CK) 18, S-100 protein P (S100-P), microtubule-associated protein 2 (MAP-2), neural cell adhesion molecule (NCAM/CD56), nestin, periodic acid Schiff (PAS) in most cases [6]. The same review found negative straining for CK 20, neurofilament (NF), CK5-6, CK7, Kir7.1, glial fibrillary acidic protein (GFAP), and epithelial cadherin (E-CAD) in most cases [6]. There are no known risk factors for PTPR [8]. Patients with pineal region tumors usually present with symptoms related to hydrocephalus and/or mass effect, including headaches (83%), vision abnormalities (67%), gait changes (41.7%), vomiting (40%), nausea, and lethargy [2,9]. Physical exams often reveal swelling of the optic disc (60%), impaired coordination (50%), impaired upward gaze (30%), upper or lower extremity tremors (20%), abnormal pupillary responses (17%), and hyperreflexia (13%) [2,9]. Initial workup for pineal region masses includes magnetic resonance imaging (MRI) of the brain and entire spinal cord, cerebrospinal fluid cytology to exclude metastatic dissemination, and blood work including alpha-fetoprotein and beta-human chorionic gonadotropin (beta-HCG) to assess whether a germ cell tumor is present (the most common type of pineal gland tumor) [2]. PTPR usually appears as a well-circumscribed heterogeneously enhancing mixed solid and cystic mass on MRI [7,9]. In 88.6% of cases, there is evidence of obstructive hydrocephalus/dilatation of the ventricle system due to aqueduct stenosis from mass extension into the third ventricle [7,9]. Initial workup and treatment often co-occur with biopsy and cerebrospinal fluid diversion to manage acute obstructive hydrocephalus if present. The most common pineal region tumors are germ cell tumors (59%), pineal parenchymal tumors (30%), and glioblastoma/astrocytoma (5%) [2]. The differential diagnosis for a pineal region mass also includes lymphoma, ganglioglioma, ependymoma, gliosarcoma, neuroendocrine carcinoma of the pineal parenchyma, schwannoma, choroid plexus papilloma, meningioma, metastases, lipomas, pineal cysts, arachnoid cysts, and vascular malformations [2,10]. Given the rarity of this malignancy, there is limited evidence to guide treatment [3]. Management approaches for PTPR include surgery, post-operative or curative RT using conventional fractionation or stereotactic radiosurgery (SRS), or observation for patients unable to tolerate treatment or with limited life expectancy / poor performance status [7,9]. Smaller tumor size and treatment with surgery (regardless of the extent of resection) were associated with improved prognosis in a meta-analysis and surgery is almost always recommended for patients with PTPR after the pathologic diagnosis is determined [7,9]. Tumor bed radiotherapy (RT) usually recommended post-operatively for all patients regardless of WHO grade, although the evidence of the benefit of this approach is inconclusive in the literature [7,9]. There are no established systemic therapy indications for PTPR [9]. PTPR has a very high risk of recurrence following surgery and post-operative RT, with a 5-year progression-free survival of only 34.5% and a 5-year overall survival of <75% [3]. In this article, we present the case of a 69-year-old man with PTPR treated with surgery and adjuvant tumor bed radiotherapy. We discuss his presenting symptoms, investigations, and management, including RT volumes and dose fractionation, and review the PTPR treatment literature.

This article was previously posted to the Preprints.org preprint server on October 8th, 2024.

## Case presentation

**Figure 1:**
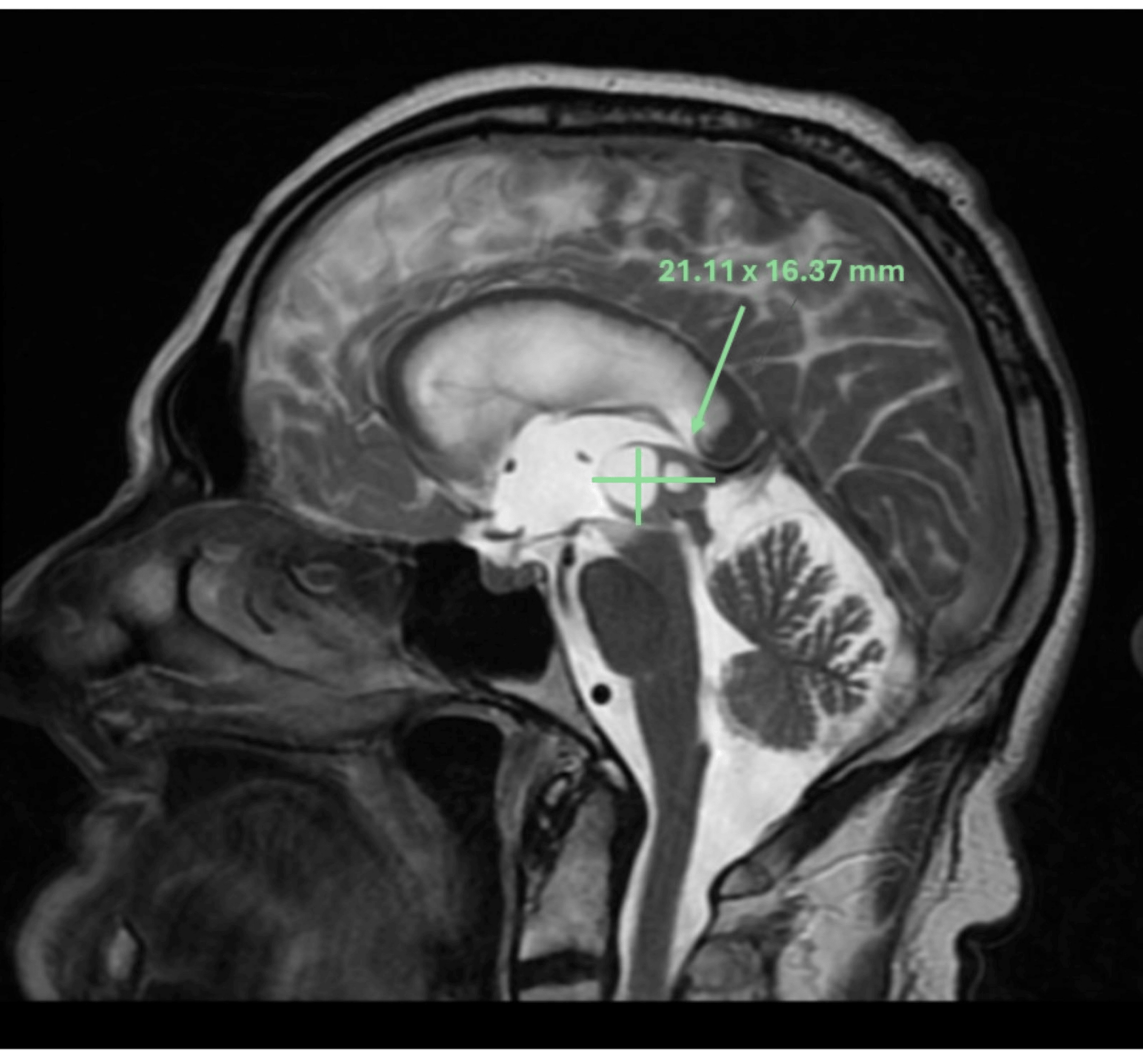
The sagittal T2 MRI head showed a pineal region mass with a hyperintense anterior cystic component.

**Figure 2:**
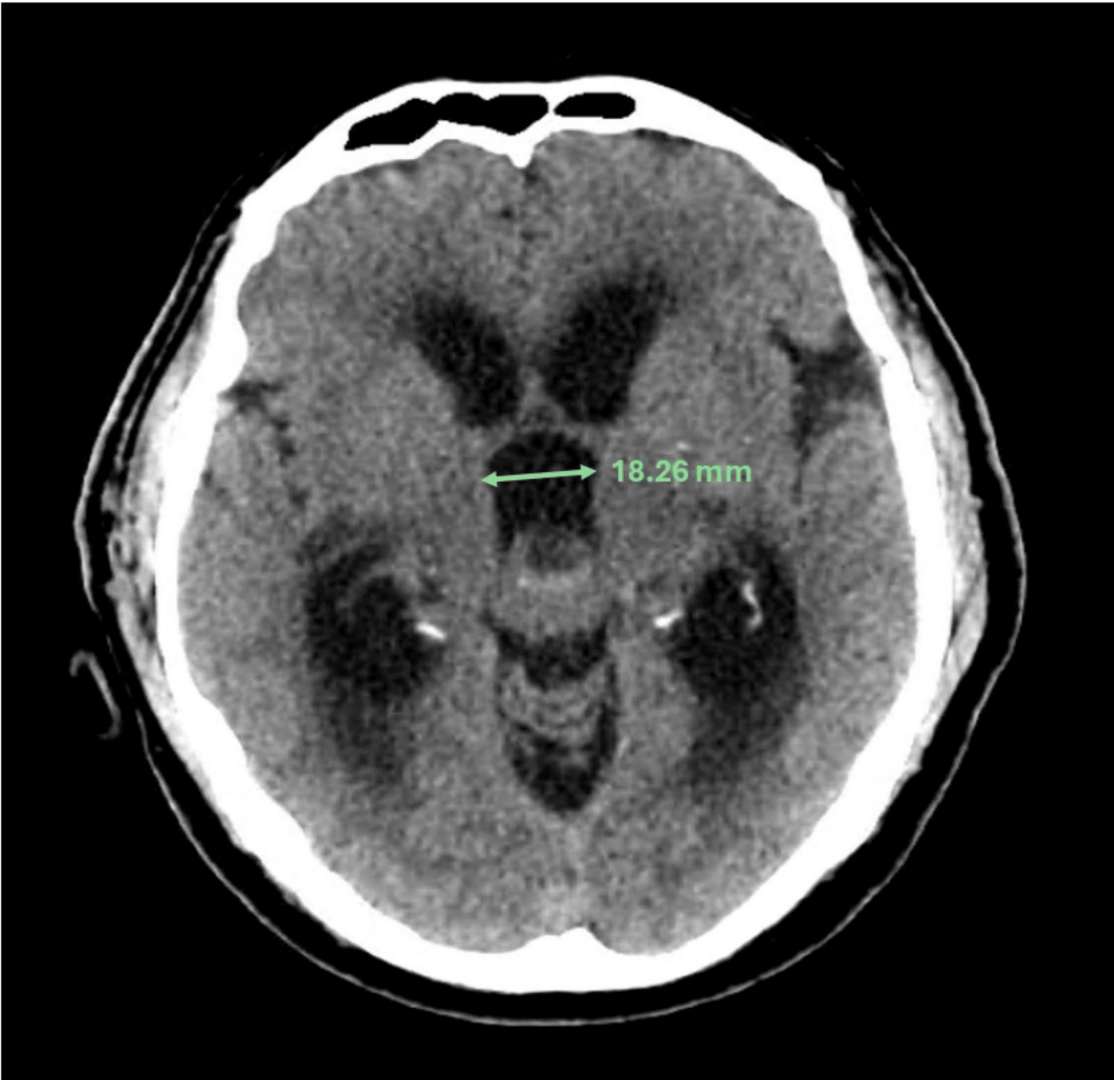
Axial CT head with hydrocephalus / third ventricle dilatation secondary to the aqueduct of Sylvius obstruction by the pineal region mass.

A man in his late 60s was brought to the emergency department by his family following two weeks of confusion, anorexia, dehydration, and gait changes. He was known for myocardial infarction with stenting of the left anterior descending artery, cervical stenosis, chronic renal insufficiency, glaucoma, well-controlled hypertension, and dyslipidemia. Prior surgeries included iridectomy, appendectomy, and umbilical hernia repair. He took low-dose aspirin, ramipril, metoprolol, dorzolamide-timolol eye drops, and Lipitor daily. He had a 45-pack-year smoking history but had quit after his myocardial infarction seven years prior and did not drink alcohol or use recreational drugs. There was no noteworthy family history. The physical exam showed altered mental status (the patient was unaware of the reason he was brought to the hospital), right cranial nerve VI palsy, anisocoria (right pupil larger than left), decreased pupillary light reflexes in the right eye, and mild gait ataxia. Vital signs and the rest of the neurologic exam were unremarkable. CT and MRI of the brain showed a 2.1 x 1.7 x 1.6 cm extra-axial mass in the pineal region with mixed solid and liquid components. A 9 mm anterior cystic component was hypointense on T1 and strongly hyperintense on T2 with post-gadolinium enhancement (Figure 1). A 7 mm posterior component of the mass was hypertense on T1, T2, and FLAIR imaging, and a hemorrhagic necrotic portion was suspected. Obstruction due to mass effect on the aqueduct of Sylvius was present, causing significant upstream hydrocephalus with third ventriculomegaly and signs of transependymal resorption with periventricular increased T2 and FLAIR signal. The third ventricle measured 4.9 x 1.8 cm (Figure 2). Foci of diffusion restriction were present, but the diffusion and SWAN studies were otherwise non-contributory. He did not have any prior imaging of the brain for comparison. The initial diagnostic hypothesis was a pineocytoma or germ cell tumor.

Complete blood count, blood glucose, electrolytes, renal and liver function tests, thyroid stimulating hormone, beta-HCG, and alpha-fetoprotein were normal. He was admitted to neurosurgery and CT chest, abdomen, and pelvis and MRI of the entire spine were negative for suspicious findings.

**Figure 3:**
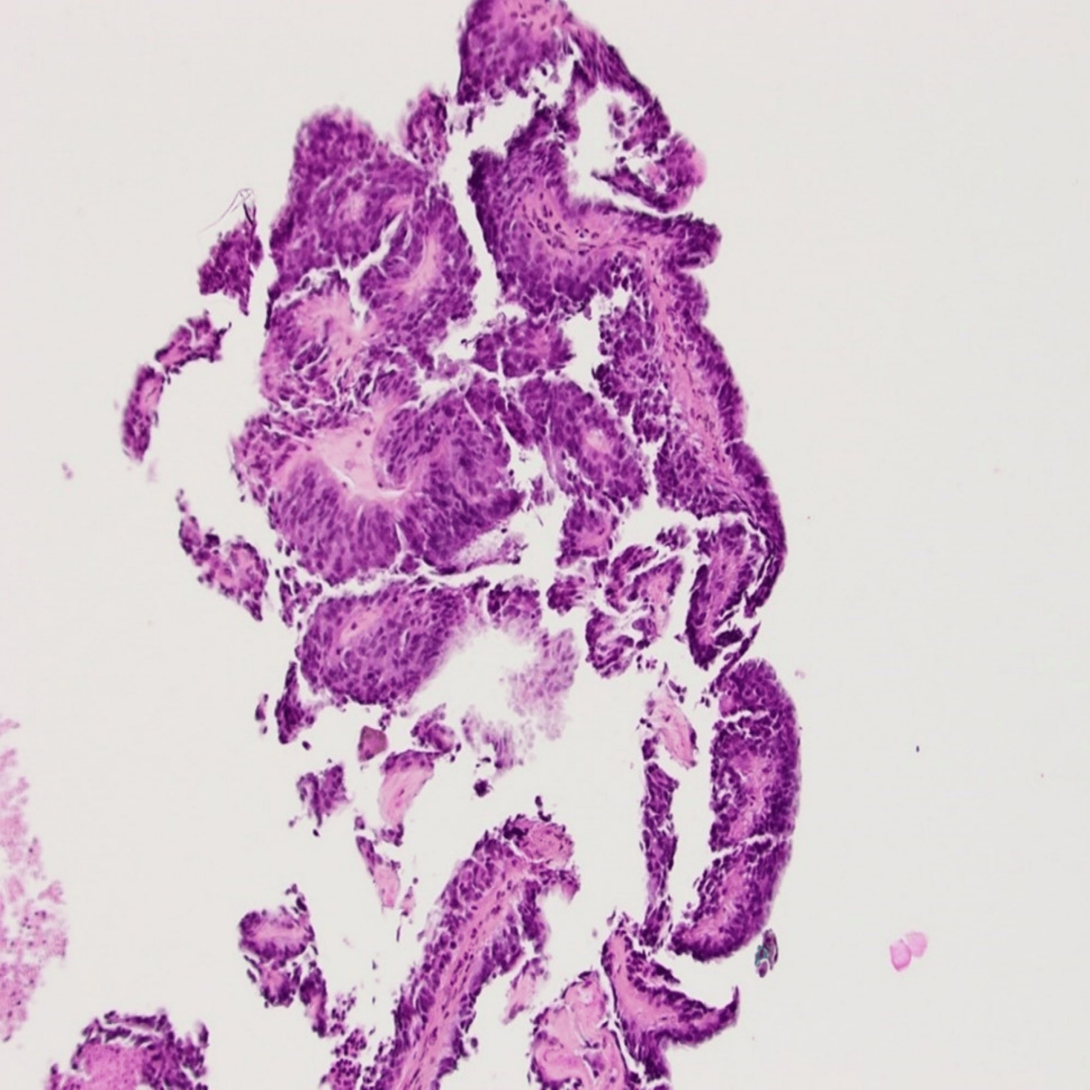
Biopsy of the pineal region mass showing cellular proliferation with epithelial appearance, forming papillae lined by tall cylindrical cells with clear eosinophilic fibrillar cytoplasm and hyperchromatic oval nuclei.

**Figure 4:**
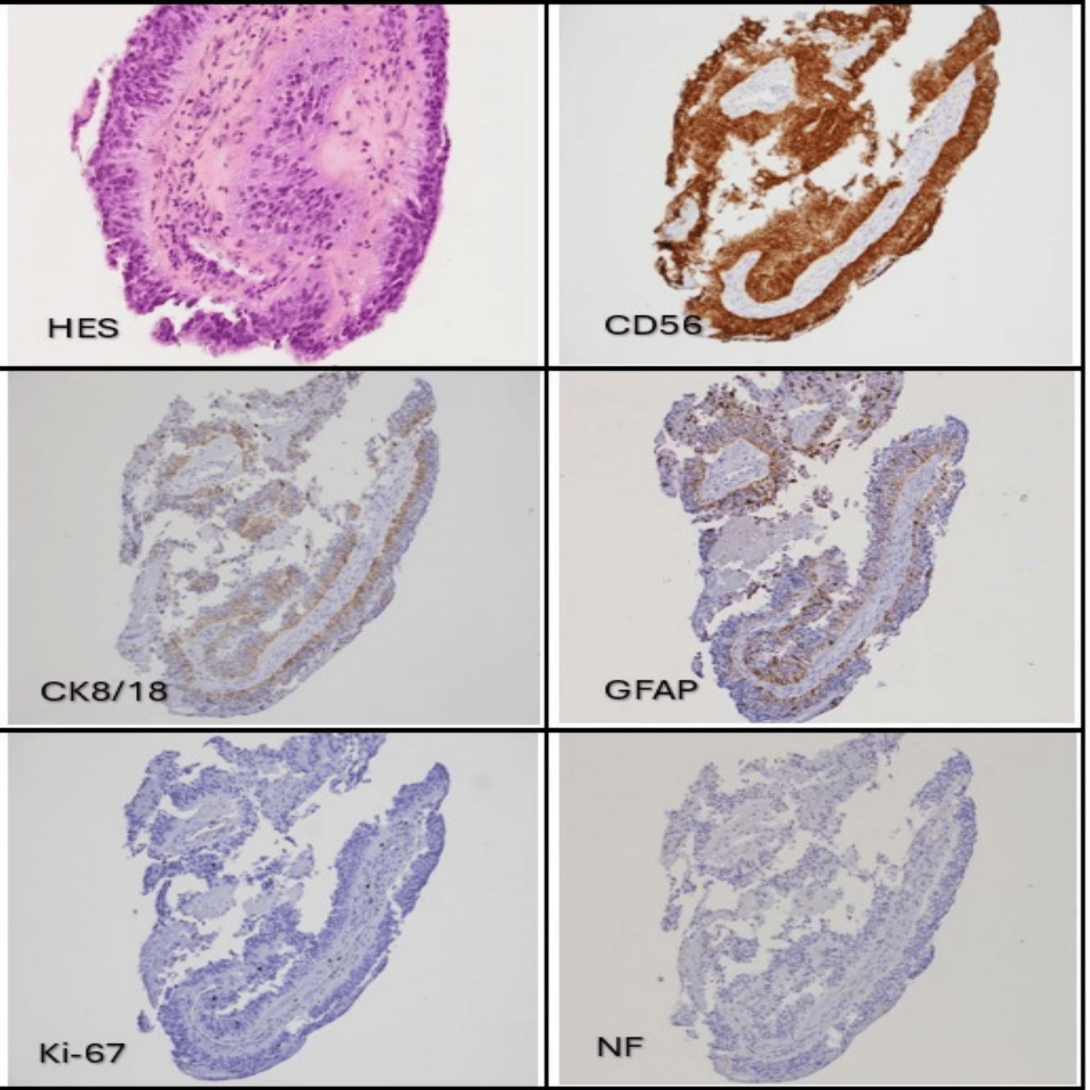
Hematoxylin and eosin staining showed the tumor's papillary structure. CD56/NCAM was strongly expressed. CK 18/8 and GFAP were focally expressed. Ki-67 was low on the initial biopsy, showing a proliferation index between 1% and 2%. This tumor was negative for NF, epithelial membrane antigen (EMA), synaptophysin, chromogranin, and E-CAD, among others.

Four days after admission, the patient became disoriented and stopped speaking (Glasgow coma scale 6). An urgent CT scan of the brain without contrast showed increased hydrocephalus with decreased high convexity sulci visibility. The patient was transferred to the intensive care unit, Diamox 50 mg po bid was started, mannitol 150 mg IV x 1 was given, and the head of his bed was elevated to 30 degrees. He was intubated and treated with urgent external ventricular drainage (EVD). Cerebrospinal fluid cytology was negative for neoplastic cells. The patient’s neurological status did not improve after EVD, and two days later, he was treated with a third ventriculostomy and biopsy of the pineal region mass. A post-operative CT scan of the head showed reduced hydrocephalus with decreased dilatation of the third ventricle and frontal horns. The patient’s neurologic status rapidly improved following ventriculostomy, and he was extubated on postoperative day 1, and the EVD was removed. Biopsy of the pineal region mass showed cellular proliferation with epithelial appearance, forming papillae lined by tall cylindrical cells with clear eosinophilic fibrillar cytoplasm and hyperchromatic oval nuclei (Figure 3). A few foci of necrosis were present, but there was no evidence of increased mitosis. The cells were strongly positive for Vimentin and CD56, focally positive for CK8/18, CKAE1/AE3, GFAP, and S100-P. The cells did not express N-ethyl-N-nitrosourea (ENU), neurofilament, synaptophysin, or E-CAD. Ki-67 was 1-2% (Figure 4). The preferred diagnosis was a papillary tumor of the pineal region, WHO grade 2 or 3. The differential diagnosis included ependymoma, choroid plexus tumor, and less likely other pineal tumors or metastasis. The diagnosis was retained following a second opinion by a neuropathologist from a quaternary hospital.

The patient was transferred to a quaternary cancer center and treated with right suboccipital craniotomy and dissection of the pineal region mass using a supracerebellar infratentorial approach. He had mild diplopia post-operatively that had been present before surgery and pain around the craniotomy site that was relieved with Tylenol. He recovered rapidly and was discharged three days after surgery. The pathology showed a proliferation of monotonous cells with solid and papillary patterns with focal hemorrhagic and necrotic zones. The neoplastic cells displayed moderately abundant cytoplasm with well-defined borders and epithelioid appearance, ovoid nuclei, and prominent nucleoli with papillary structures around the vessels suggestive of perivascular pseudorosettes. The tumor cells were highly expressive of MAP2, CD56, NSE, and CK18/8 around the papillary structures. C-MYC antibody was positive in most nuclei, and S100-P was weakly positive in a subpopulation of cells. The mitotic index was 2-3/mm2, and the Ki-67 was up to 15-20% in certain foci. NEUN, NF, synaptophysin, chromogranin, CD45, BRG1, GFAP, P53, IDH1 R132H, ATRX, OLIG2, BRAF V600E, CD34, EMA, E-CAD, and SMARCB1/INI1 were not significantly expressed. The pathology was consistent with a diagnosis of papillary tumor of the pineal region, WHO grade 3. The solid tumor panel (Ampli focus panel) was negative for clinically relevant DNA, RNA, or copy number variation (CNV) alterations. The patient’s case was presented at neuro-oncology tumor board, and the consensus was to treat with adjuvant radiotherapy. The patient was treated with external beam radiotherapy to a dose of 54 Gray (Gy) in 30 once-daily fractions to the tumor bed. Radiotherapy consisted of 6 MV photons, was planned using Varian Medical Systems' Eclipse 16.1 software (Palo Alto, CA, USA) and delivered using a volumetric modulated arc therapy (VMAT) technique on a Clinac iX linear accelerator (Varian Medical Systems, Palo Alto, CA USA).

Table 1 outlines the adjuvant RT planning volumes and dose fractionation used. The standard organ at-risk radiation dose constraints for conventional fractionation were respected (Table 2) [10-12].

**Table 1:** Radiotherapy treatment volumes and dose fractionation. GTV, gross tumor volume; CTV, clinical target volume; PTV, planning target volume; Gy, Gray; CT, computed tomography; MRI, magnetic resonance imaging; pre-op, preoperative; post-op, post-operative. *Anatomic boundaries included brain parenchyma that the tumor had displaced.

Treatment Parameter	Definition
GTV	Residual disease based on pre- and post-op imaging
CTV	GTV + tumor bed on planning CT and MRI + 5 mm margin cropped to anatomic boundaries*
PTV	CTV + 5 mm concentric margin
Dose and fractionation	54 Gy / 30 fractions delivered Monday to Friday over six weeks in 1.8 Gy once daily fractions

**Table 2:** Organ at risk radiation dose constraints (conventional fractionation*) V54Gy, the volume of the organ at risk receiving 54 Gy or more; Gy, Gray; cc, cubic centimeter; mm, millimeter; V45Gy, the volume of the organ at risk receiving 45 Gy or more; V60Gy, the volume of the organ at risk receiving 60 Gy or more. *Dose constraints for RT delivered in 1.8-2 Gy, once daily, fractions

Organs at risk	Dose constraint (to organ at risk + 3 mm concentric margin)
Brainstem	V54 Gy < 0.03 cc
Spinal cord	V45 Gy < 0.03 cc
Optic chiasm	V54 Gy < 0.03 cc
Optic nerves	V54 Gy < 0.03 cc
Brain	V60 Gy < 33%, V50 Gy < 66%
Eyes	V45 Gy < 0.03 cc

**Figure 5:**
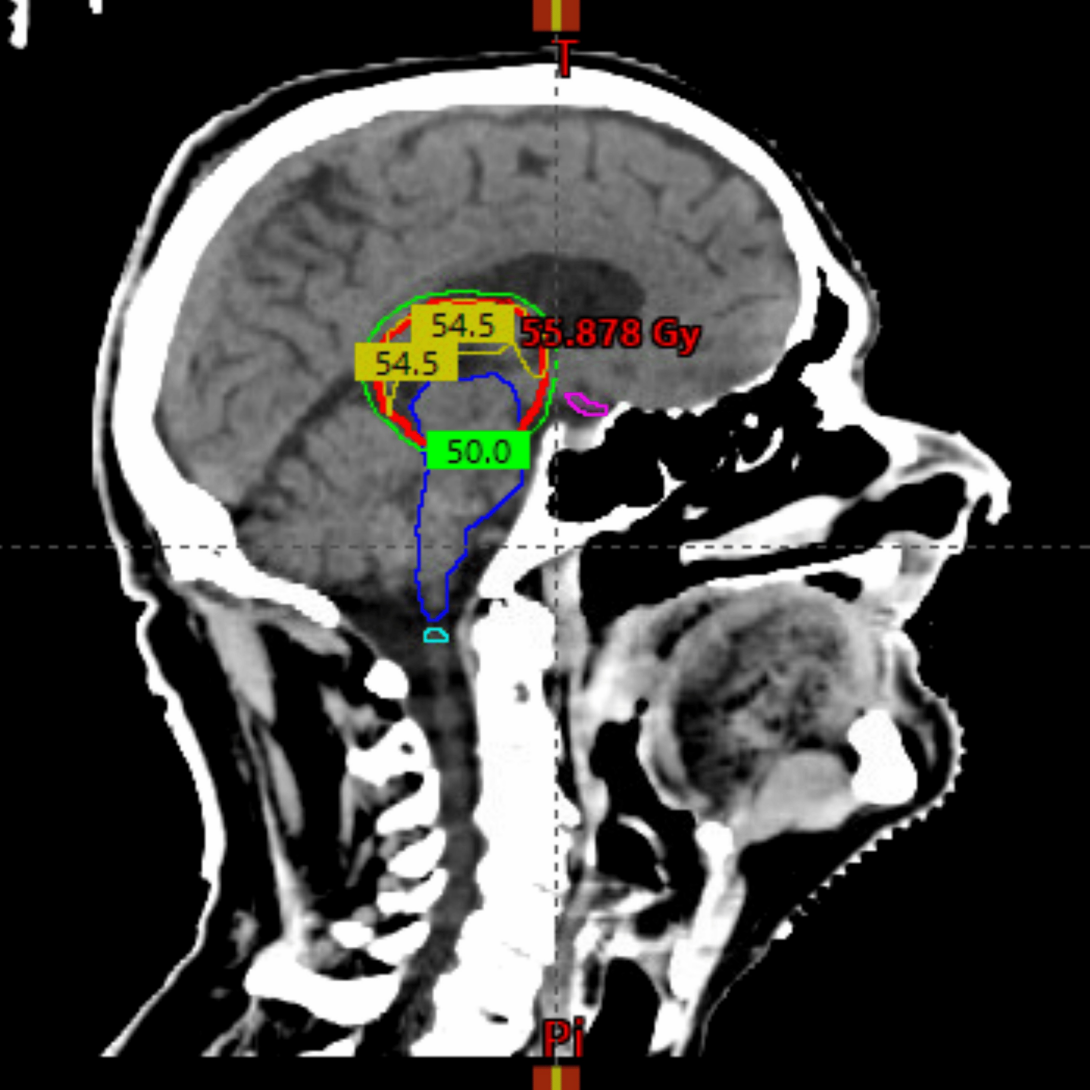
A sagittal view of the planning CT scan shows the 50 Gy isodose line (green) covering the PTV (red). The 54 Gy isodose line (yellow) curves superiorly to respect the brainstem (blue) and optic chiasm (magenta) radiation dose constraints. PTV, planning target volume

The treatment planning objective for the planning target volume (PTV) coverage was V50GY > 99% (see Figure 5 below).

After adjuvant RT, the patient was followed with a contrast-enhanced brain MRI every three months. At 18 months post-treatment, he reported no symptoms, and the MRI was negative for recurrent disease. A CARE checklist for this case is included in the supplementary materials (see Appendices).

## Discussion

PTPR is an exceedingly rare malignancy without randomized controlled trials to guide treatment [3]. Patients are diagnostically challenging because they usually present with neurologic symptoms caused by obstructive hydrocephalus, and investigations and initial treatment happen concurrently [2]. Access to neurosurgery and radiation oncology care is not widely available outside of major cities, and patients living far away from tertiary care hospitals face additional access and financial burdens associated with having to travel, often very long distances, for treatment [13]. Our review of the literature identified two systematic reviews, four review articles, one multicenter study, and seven case reports. We also identified seven articles that describe treatment volumes for adjuvant conventionally fractionated radiotherapy (Table 3). Yamaki et al. systematically reviewed the literature and identified 71 studies with 177 patients with PTPR [7]. They found that 36-month overall survival was improved when surgery was a part of treatment (hazard ratio (HR) 0.16, 95% confidence interval (CI) 0.05-0.45, p = 0.001) [7]. The only other significant prognostic factor was tumor size, with each additional centimeter associated with worse survival (HR 1.99, 05% CI 1.12-3.53 p=0.19) [7]. No additional benefit was found for gross total resection (versus subtotal resection or biopsy), adjuvant radiotherapy, or systemic therapies [7]. Another systematic review by Lancia et al. included 26 studies with 116 PTPR patients and reported that the majority of patients (52.6%) obtained gross total resection, and most patients (72.4%) received radiotherapy as part of their therapeutic strategy [9]. About 70% of patients treated with radiotherapy received adjuvant radiotherapy with 50.4-60 Gy in 1.8-2 Gy per fraction, the most common dose fractionation. About 28.6% of patients were treated with curative radiotherapy, mostly with Gamma Knife stereotactic radiosurgery (SRS), using doses ranging from 12-36 Gy in 1 fraction, although conventional fractionation was also used for curative treatment [9]. Focal irradiation to the tumor bed was used in most cases, but whole brain or craniospinal irradiation with boost was also used in 9.8% of cases. About 18.5% of patients received adjuvant chemotherapy using combinations of temozolomide, vincristine, cisplatin, etoposide, nimustine, and ifosfamide [9]. The authors did not analyze the impact on local control or survival associated with each treatment modality. A multicenter study by Fauchon et al. of 44 patients with histopathologically proven PTPR found that gross total resection was significantly associated with increased overall survival compared to subtotal resection or biopsy only (p=0.04) and that older age was associated with worse overall survival (p=0.03) [14]. The authors found no overall survival or progression-free survival benefit from adjuvant radiotherapy or chemotherapy [14]. Surgery is almost always recommended for PTPR after the pathologic diagnosis is confirmed and based on the available literature, is the most evidence-based intervention [7,9]. After surgery, adjuvant radiotherapy (RT) is also usually given despite the lack of evidence of benefit and most often consists of external beam radiotherapy to the tumor bed and macroscopic disease with doses of 45-59.4 Gy in 1.8-2 Gy once daily fractions [7,9,14-20].

**Table 3:** Publications reporting adjuvant RT treatment volumes for PTPR #, number; adj, adjuvant; RT, radiotherapy; GTR, gross total resection; STR, subtotal resection; GTV, gross tumor volume; CTV, clinical target volume; Gy, Gray; NA, not available.

Author	Year	Publication Type	# Patients	Surgery Type	Adj RT Dose/Fractionation	RT Volumes
Buffenoir et al. [16]	2008	Case Report	1	GTR	50 Gy / 1.86 Gy per fraction. Five fractions per week	"Tumor bed plus wall of third ventricle"
Epari et al. [17]	2011	Case Report	2	GTR (both patients)	54 Gy / 30 fractions (both patients)	"Local radiation"
Lancia et al. [9]	2017	Case Report	1	Recurrent disease. STR	59.4 Gy / 33 fractions. Five fractions per week	CTV = contrast-enhanced lesion + 5 mm margin, PTV = 3 mm expansion of CTV
Choque-Velasquez et al. [18]	2018	Case Report	1	GTR	54 Gy divided into daily doses of 1.8 Gy	"Focal RT to the pineal tumor bed"
Pons-Escoda et al. [19]	2022	Case Report	1	STR	50 Gy / 25 fractions	"Enhancing area (on post-op MRI)"
Lombardi et al. [3]	2022	Review Article	NA	RT indicated for STR or recurrent tumors	50.4-54 Gy in 1.8-2 Gy fractions	“Focal RT”
Mesny and Lesueur [20]	2023	Review Article	NA	RT indicated for STR or recurrent tumors	50.4-54 Gy in 1.8-2 Gy fractions	GTV = post-operative cavity and any residual lesion. CTV = margins are undefined

Other treatment strategies including whole brain radiotherapy and craniospinal irradiation are also sometimes used, particularly in cases with spinal dissemination, with doses of 30.4-36 Gy to the craniospinal axis with an 18-23.4 Gy boost to the tumor bed and gross disease [3,7,9,14-15]. Curative RT is reserved for cases where surgery isn’t possible [3,7,9]. Stereotactic radiosurgery (SRS) with doses ranging from 12-36 Gy in 1-3 fractions has also been used in curative and adjuvant settings with good local control and low toxicity [7,9]. Publications using brachytherapy are limited, but interstitial brachytherapy with Iodine-125 in the adjuvant setting has been reported [21]. Adjuvant chemotherapy is also used sometimes (18.5% in the systematic review by Lancia et al.) but the two available publications that attempted to quantify the impact reported no benefit [7,9,14]. Toxicity rates following treatment are reassuringly low. Out of 116 patients with PTPR treated with surgery and RT analyzed by Lancia et al., only three developed adverse effects, including one patient with grade 2 fatigue, one with grade 3 diplopia and hypersomnia, and another with grade 3 motor deficiency and Parinaud syndrome [9]. PTPR has a very high risk of local recurrence despite aggressive treatment that can be greater than 80% at 10 years [3,9]. The European Reference Network for all rare adult solid cancers (EUROCAN) reports an overall survival rate of < 75% at five years [3]. Recently published data by the National Cancer Institute highlights that PTPR is a heterogeneous disease and that molecular characteristics such as DNA methylation and genomic copy number differences confer prognostic implications for clinical outcomes [22]. Innovative treatment approaches include pharmacotherapy for patients with PTPR with specific mutations, including a patient with PTPR with PTEN R130Q chromosome 10 loss treated with everolimus monotherapy who experienced continuous tumor regression over 27 months and had been stable for 43 months at the time of publication [23].

## Conclusions

PTPR is a rare central nervous system malignancy without clinical trials to guide treatment. The most common treatment is surgery, followed by postoperative tumor bed RT with 45-59.4 Gy / 25-33 fractions. Based on the available literature, smaller tumor size and treatment with surgery might be associated with better outcomes. The evidence for the benefit of other treatment modalities is mixed. Following treatment, recurrence rates are very high, and further research is needed to improve outcomes.

## Figures and Tables

**Figure 6:**
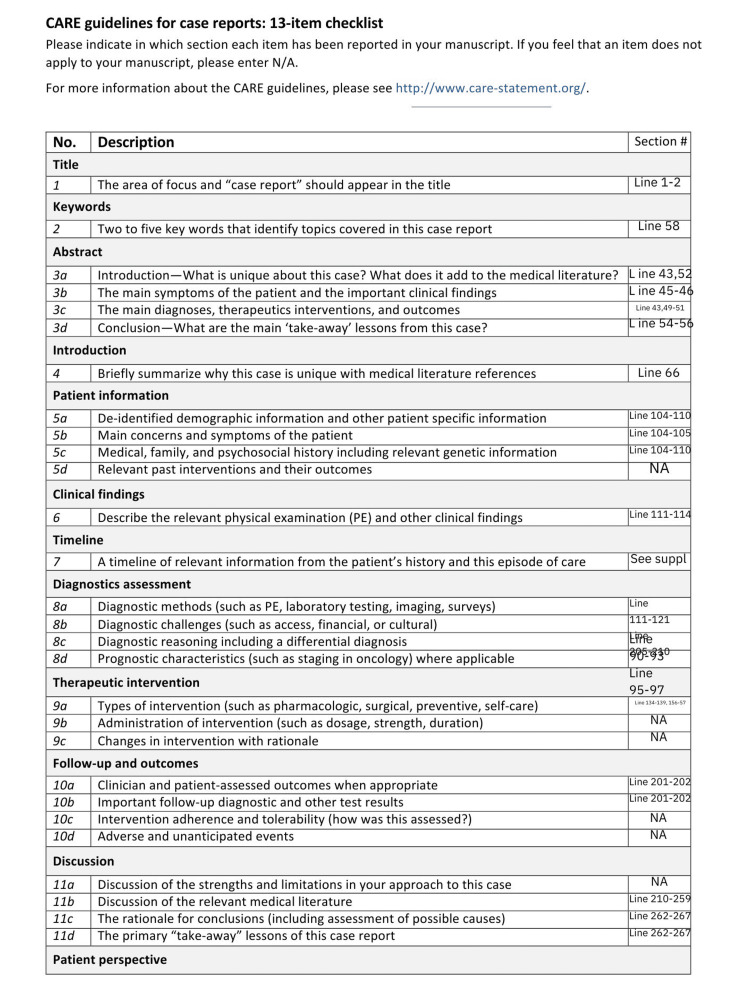
CARE Checklist 1/2

**Figure 7:**
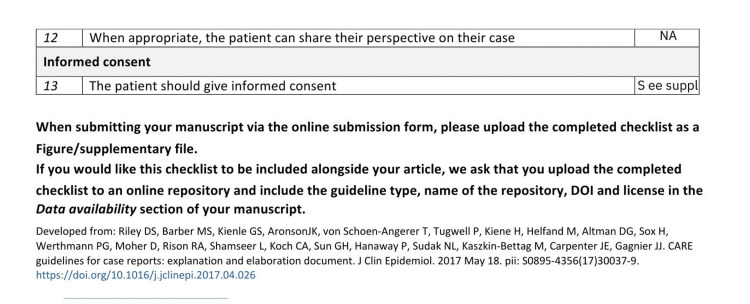
CARE checklist 2/2
